# A Pilot Study for Evaluation of Digital Systems as an Adjunct to Sphygmomanometry for Undergraduate Teaching

**DOI:** 10.7759/cureus.736

**Published:** 2016-08-15

**Authors:** Shival Srivastav, Renuka Sharma, Raj Kapoor

**Affiliations:** 1 Physiology, Vardhman Mahavir Medical College and Safdarjung Hospital

**Keywords:** blood pressure, sphygmomanometer, small group learning

## Abstract

Objectives: Blood pressure estimation is a key skill for medical practitioners. It is routinely taught to undergraduate medical students using an aneroid sphygmomanometer. However, the conceptual understanding in the practical remains limited. We conducted the following study to evaluate the efficacy of digital data acquisition systems as an adjunct to the sphygmomanometer to teach blood pressure.

Methods: Fifty-seven first-year medical students participated in the study. An MCQ test of 15 questions, consisting of 10 conceptual and five factual questions, was administered twice – pre- and post-demonstration of blood pressure measurement using a digital data acquisition system. In addition, qualitative feedback was also obtained.

Results: Median scores were 7 (6 - 8) and 3 (1.5 - 4) in pre-test sessions for conceptual and factual questions, respectively. Post-test scores showed a significant improvement in both categories (10 (9 - 10) and 4 (4 - 4.5), respectively, Mann-Whitney U test, p < 0.0001). Student feedback also indicated that the digital system enhanced learning and student participation.

Conclusions: Student feedback regarding the demonstrations was uniformly positive, which was also reflected in significantly improved post-test scores. We conclude that parallel demonstration on digital systems and the sphygmomanometer will enhance student engagement and understanding of blood pressure measurement.

## Introduction

Blood pressure measurement is one of the most common procedures performed by health care providers, which gives important information about the patient's overall health status. However, this simple procedure is very often executed improperly, producing inappropriate readings that can lead to over- or under-treatment of a patient [1-2].

Hence, medical students are trained in this skill from the first year onwards as part of the physiology practical curriculum. In most medical schools, the conventional Riva-Rocci/Korotkoff’s technique of measuring blood pressure with a mercury sphygmomanometer and stethoscope is being followed. However, blood pressure measurement is a complex skill that requires considerable practice in order to gain competence in the recognition of Korotkoff sounds. Interactions with our medical students revealed that the conceptual understanding of the blood pressure practical was far from satisfactory in the majority. Hence, the competency of blood pressure estimation attained by the students was more of a skill rather than the underlying concept. Previous studies have also demonstrated that common errors during blood pressure measurement, like placing stethoscope diaphragm under the cuff, persisted in fourth and fifth-year medical students as well [3].

Certain researchers have attempted to clarify the concept of blood pressure measurement using simulators and supplemental classes to nursing students [4-5]. To the best of our knowledge, no such study has been carried out in medical students to enhance their understanding of the physiological phenomena underlying blood pressure estimation.

Hence, we designed this study to explore demonstration on digital data acquisition systems, as a supplemental tool, for teaching blood pressure recording to first-year medical students.

## Materials and methods

After due approval by Institute Ethics Committee, the study was launched by explaining a brief outline to all the first-year medical students during lecture hours. All subjects who volunteered for the study were enrolled in a random manner and the identity of the students was coded to preserve anonymity and avoid bias. All the non-participants were also assured of no adverse action for refusal to participate.

After obtaining informed consent, the study protocol was explained and students were distributed in groups of four to five each. Assessment of baseline knowledge was done using a multiple choice question (MCQ) test, which consisted of 15 questions: 10 conceptual and five factual questions (See Appendix I).

The questions were prepared from standard undergraduate textbooks and consisted of four options each. Subsequently, demonstration of blood pressure estimation was done using PowerLab® digital data acquisition system (AD Instruments, New South Wales, Australia). To ensure uniformity of demonstration, all sessions were conducted by the same person and a standard script was read out. After the demonstration, the MCQ test was re-administered to the participants. In addition, a questionnaire to document qualitative feedback was also given.

### Demonstration using a digital data acquisition system

The PowerLab®​ digital data acquisition system  was used to conduct the demonstration. The system is already being used for research worldwide and data acquired using it has been validated in multiple scientific publications [6].

We used three channels of the device for demonstration – a pressure transducer attached to Riva-Rocci cuff, cardio microphone, and pulse transducer, respectively. The pressure transducer was appropriately calibrated before the start of every demonstration.

**Figure 1:**
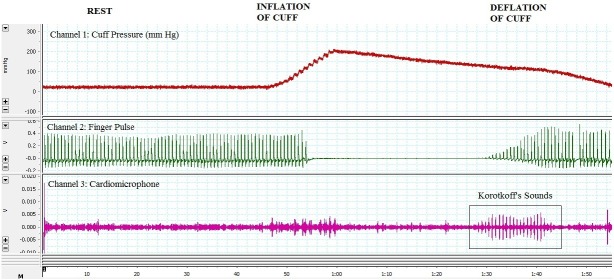
Representative Record of Blood Pressure Measurement Using the PowerLab© System This is a three channel recording done using PowerLab® system for estimation of blood pressure. Channel 1 shows pressure in Riva-Rocci cuff taken using pressure transducer. Channel 2 shows pulse waveform taken using pulse transducer from the index finger of ipsilateral arm. Channel 3 shows sounds picked up using a cardio microphone. The phases of rest, inflation of cuff and deflation of cuff are marked. During rest, the cuff pressure is close to zero, finger pulse is normal in amplitude, and cardio microphone channel shows some noise due to mechanical pulsations picked up by the probe. As the cuff is gradually inflated, the cuff pressure rises and stray signals are picked up by the sensitive microphone due to turbulent flow. When cuff pressure surpasses systolic pressure, finger pulse completely disappears. When the cuff is slowly deflated, finger pulse reappears and gradually rises in amplitude and the Korotkoff sounds can be clearly demarcated in Channel 3.

The demonstration began by tying the Riva-Rocci cuff around the arm, placing a cardio microphone over the brachial artery, and strapping the pulse transducer to the index finger of the ipsilateral arm. As the cuff was gradually inflated, the amplitude of the finger pulse began diminishing. At the point of disappearance of the pulse, the pressure in the cuff was taken as a measure of systolic blood pressure. Cuff pressure was inflated to 10-16 mmHg beyond this point and subsequently deflated slowly. As the pressure approached systolic pressure, finger pulse reappeared and Korotkoff sounds were observed in the microphone channel. As the pressure was further lowered, finger pulse regained its normal amplitude and Korotkoff sounds disappeared when pressure attained diastolic levels, as seen in Figure 1.

### Assessment of MCQ score

The MCQ test administered consisted of two parts. The initial 10 questions were based on physiological concepts underlying measurement of blood pressure using a sphygmomanometer while the latter five questions were factual and dealt with commonly used terminologies, such as Reynold’s number, white coat hypertension, etc.

As discussed earlier, the MCQ test was administered pre- and post-demonstration and scores were compared for conceptual and fact-based questions. One mark was awarded for a correct answer while no marks were awarded for incorrect answers or unattempted questions. The individual scores for both categories were compared pre- and post-demonstration.

### Qualitative assessment

Along with the post-demonstration MCQ test, a questionnaire with three open-ended questions was administered to the students (See Appendix II).

The questions recorded the perception and suggestions of the students regarding the utility of the demonstration via the digital data acquisition system.

### Statistical analysis

GraphPad Prism® software version 5.01 (GraphPad Software, San Diego, CA) was used for statistical analysis. Kolmogorov-Smirnov test was used to assess normality of distribution. Data was expressed as median (interquartile range). Mann-Whitney U-test was used to compare pre- and post-demonstration score for both categories of questions.

## Results

Fifty-seven medical students participated in the study. Marks were awarded for concept-based and factual questions answered correctly out of 10 and five, respectively.

**Figure 2:**
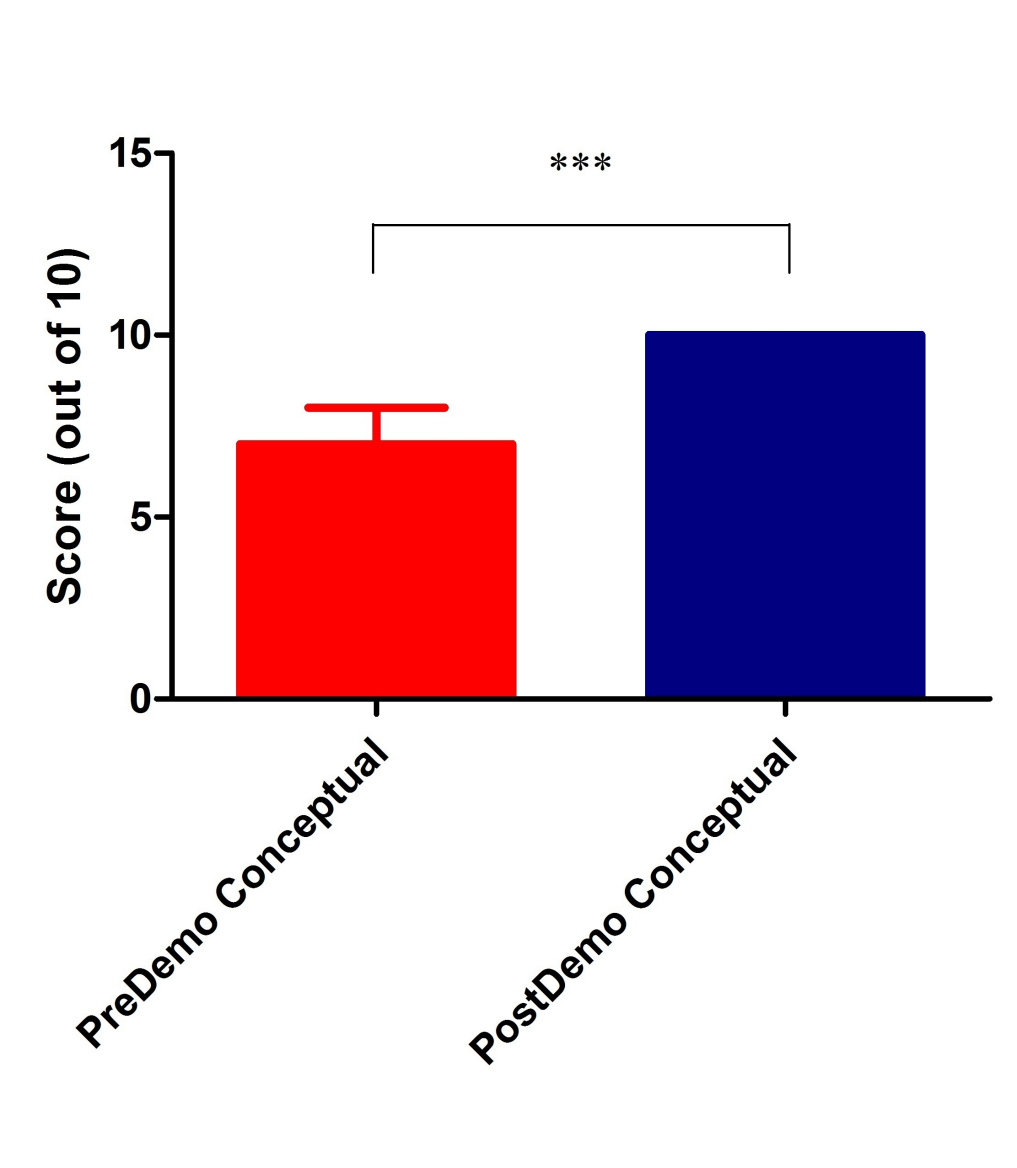
Comparison of Pre- and Post-test Scores for Conceptual Questions (n = 57) Mann-Whitney U test, p < 0.0001

**Figure 3:**
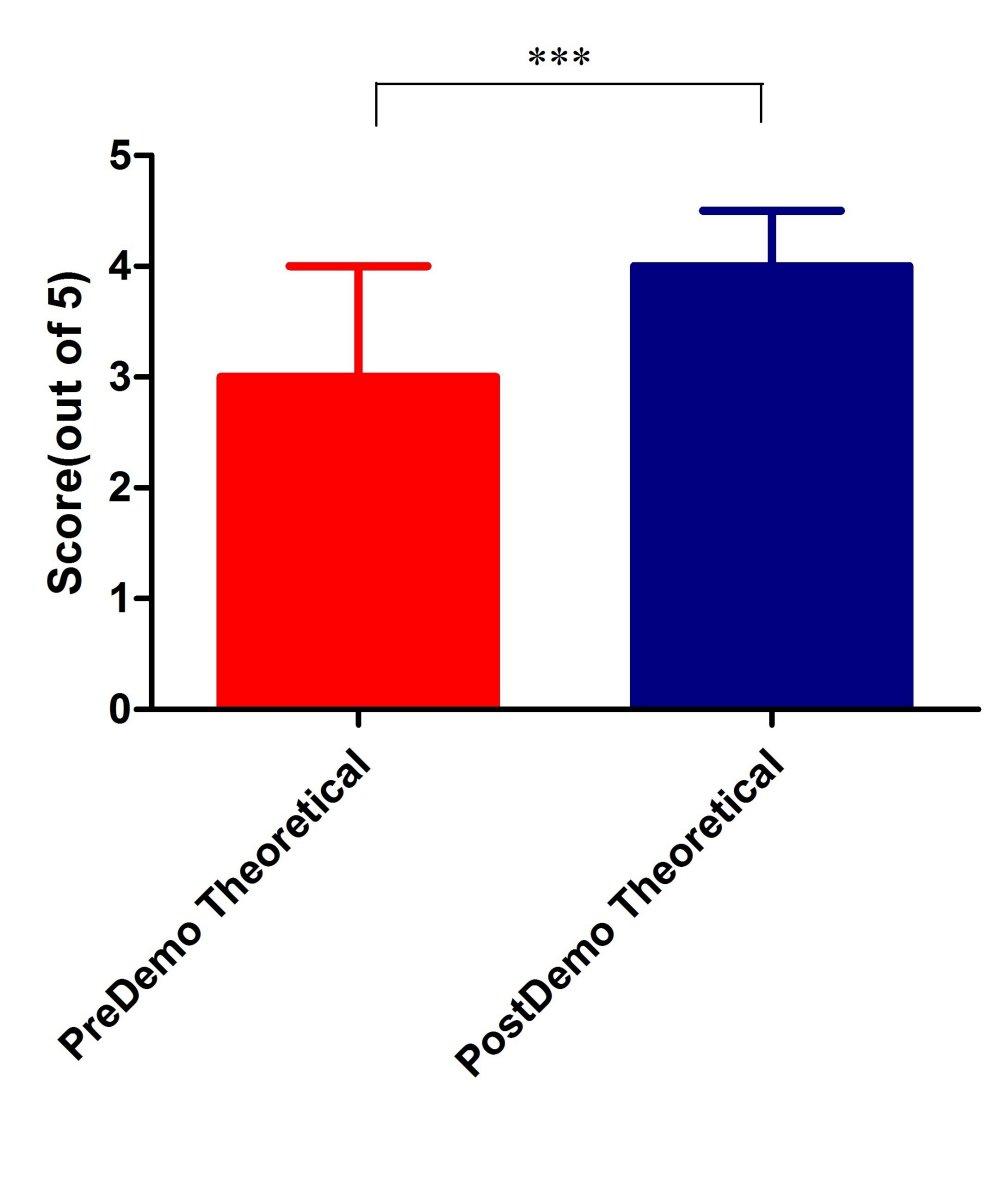
Comparison of Pre- and Post-test Scores for Theoretical Questions (n = 57) Mann-Whitney U test, p < 0.0001

Data was expressed as median (Interquartile range). The median score for conceptual and factual questions were 7 and 3, respectively, in pre-test scores. Post-test scores showed a significant improvement in both categories (10 (9-10) and 4 (4-4.5), respectively, as seen in Table 1 and Figures 2-3 below, (Mann-Whitney U test, p < 0.0001).

**Table 1:** Comparison of Scores for Conceptual Questions and Theoretical Questions (n = 57) *Data expressed as median (interquartile range)

Question Type	Pre-Test Score*	Post-Test Score*	P value
Conceptual (Maximum marks = 10)	7 (6-8)	10 (9-10)	< 0.0001
Theoretical (Maximum marks = 5)	3 (1.5-4)	4 (4-4.5)	< 0.0001

Most students reported that the blood pressure demonstration enhanced their understanding of the practical and helped them to understand its basis better. From the participants’ viewpoint, they gained more satisfaction via interaction with the faculty in a small group during the demonstration, which helped them to raise doubts and clarify their concepts.

On being asked to describe their first exposure to a digital data acquisition system, all students expressed a positive opinion regarding the system and its usefulness for better comprehension. In fact, most students expressed their desire to work on the system and record the blood pressure independently.

One participant said that “The demonstration was interesting and useful and enhanced my understanding of the topic” (male first-year student)

Another reported that “I was finally able to understand how Korotkoff sounds are produced and the phenomenon of an auscultatory gap” (male first-year student).

When asked how the demonstration was different from using the aneroid sphygmomanometer, a majority of participants found it to be visually illustrative and easier to comprehend as compared to sphygmomanometry.

One participant mentioned that “The demonstration was more descriptive” while another student wrote that “I could visualise the Korotkoff sounds better using this method” (female first-year student).

Another student found “The experience was very useful and more informative” (male first-year student).

The participants were also asked as to how the demonstration could be improved to make it more beneficial for understanding. Most students felt that holding the demonstration simultaneously with the sphygmomanometry practical would be extremely useful for learning purposes. Also, all participants strongly appreciated the benefits of small group teaching and felt it should also be carried out routinely.

One student reported that “Making it a part of the regular practical curriculum and demonstrating it along with the sphygmomanometer method would be helpful” (female first-year student).

Another student also felt that “Since this demonstration was held in small batches, we could interact and clarify our doubts better” (female first-year student).

One student said that “The demonstration would have been better if the students were allowed to take the blood pressure using this method themselves” (male first-year student).

## Discussion

Cardiovascular physiology practicals are a vital segment of the curriculum and form the basis for consolidation of theoretical concepts, the development of clinical examination skills, and are crucial in student understanding as they provide opportunities for active learning. However, most students find certain concepts of cardiovascular physiology quite challenging to fathom [7].

The use of a sphygmomanometer for teaching measurement of blood pressure to undergraduates has been in practice since time immemorial. Students are taught to record blood pressure using both the palpatory method and auscultation of Korotkoff sounds by means of a stethoscope. The principle behind the generation of these sounds is the turbulence of blood flowing through a partially occluded area in the artery. The Korotkoff sounds are used mainly to detect the values of systolic blood pressure and diastolic blood pressure, which is done by noting the levels at which the Korotkoff sounds initially appear and then disappear, respectively. However, a consistent observation during subsequent practical assessments was the difficulty in the perception of sounds and understanding of the concept of turbulent and streamlined flow faced by many students. An attempt has been made for nursing students to improve their understanding of blood pressure measurement by using simulators [4]. Also, similar conceptual doubts have been documented in medical students in various facets of cardiovascular physiology. Newer teaching methodologies, like Finapres®, ultrasound, and equivalent electronic circuits, have been used to resolve these doubts [8-12]. These modalities have been observed to be more effective aids for student engagement as they enhance their participation level and provide motivation for further learning and comprehension in an enjoyable manner. Hence, we designed this study to assess the efficacy of demonstration on a digital data acquisition system, as an aid to blood pressure measurement by the sphygmomanometric method, for first-year undergraduate students.

The study was conducted after lectures on cardiovascular physiology had been delivered and blood pressure estimation using a sphygmomanometer had been taught in multiple practical classes*.* The students had been given the opportunity of clarification of doubts during the discussion sessions conducted following the practical. Hence, it was safely assumed that the students had reasonable knowledge about the practical, which was reflected in the above average performance in the pre-test score, as seen in Table 1.

We used PowerLab® digital data acquisition system for demonstration of blood pressure recording. The demonstration displayed a correlation of pulse amplitude and Korotkoff sounds with changes in cuff pressure and was carried out in small groups of four to five students for better visual clarity and understanding. A questionnaire consisting of 15 multiple choice questions (10 conceptual and five fact-based) was prepared from standard physiology textbooks by senior faculty members with adequate expertise in undergraduate assessment. Questionnaires were administered before and after the demonstration to assess the baseline knowledge and improvement in understanding, respectively. Qualitative assessment was also recorded using open-ended questions to record the participants' perception and suggestions regarding the demonstration.

Analysis of post-test scores revealed a significant improvement in test scores, as seen in Table 1. The positive results reflect the value of a demonstration of digital data systems in the improvement of student understanding. Most participants exhibited average scores in the pre-demo test, which only confirms the well-established adequacy of sphygmomanometry as a teaching tool for undergraduates. However, the improved scores following the demonstration are an indicator of enhanced comprehension as they were able to visualize the key vascular changes and clarify their concepts regarding Korotkoff sounds.

The participants’ feedback regarding the demonstration was extremely positive, and the overwhelming majority of students strongly endorsed the concept of this demonstration to be included in the routine curriculum. Most students were vocal about the improvement in their understanding levels and clarity of concepts and also suggested that the demonstration should be held concurrently with the sphygmomanometric method for optimal comprehension.

An additional observation was the preference of most students for small group teaching as it encouraged them to raise queries and seek clarifications in problem areas. Previous studies have also observed that small group teaching is a more effective tool and promotes better learning amongst students [13-16].

Hence, we propose that digital data systems should be increasingly employed, along with the sphygmomanometer, for teaching blood pressure measurement to undergraduates as they enhance student participation and facilitate learning.

### Limitations 

Our study had a few limitations. Since the participation was purely voluntary, our pre-test scores could have been influenced by sampling bias. Students who were highly motivated and, hence, academically better performers were more likely to participate in the study and consequently score better. We tried to overcome this bias by making multiple formal announcements and encouraging all students to participate in the study. We had committed to the participation of thirty students since this was a pilot project, but eventually fifty-seven students participated in our study. The enthusiastic participation was encouraging and confirms the interest generated by novel teaching methods in students. Nonetheless, it is natural that further studies on larger samples, such as entire classes, will firmly help establish the role of digital data systems for routine undergraduate teaching.

### Strengths

To the best of our knowledge, ours was the first study of its kind that has validated the use of digital data acquisition systems for teaching the underlying basis of measurement of blood pressure to undergraduate students. Significant improvement in post-test scores reaffirmed our belief that there are some lacunae in students’ understanding of physiological principles behind seemingly simple skills, such as blood pressure estimation. These doubts may be addressed by means of new teaching methodologies, such as the digital data acquisition systems. The qualitative feedback illustrated that small group learning and newer methodologies are likely to enhance student participation and improve learning.

## Conclusions

To conclude, we observed that the introduction of a pilot demonstration practical using the digital data acquisition system at our institution was potentially beneficial for the first-year medical students. As compared to finger plethysmography and ultrasound used in prior studies, the digital data acquisition system is a highly effective and economical teaching aid, which requires no specialized training. Numerous earlier studies and our own results demonstrate that practical classes that involve active learning and student-centric approaches can significantly enhance student engagement and learning [13-17].

## Appendices

Appendix I: Multiple Choice Question Test

1. Above which Reynold’s number, will the blood flow become turbulent?
  1. 500
  2. 1000
  3. 2000
  4. 3000
2. The sounds heard during estimation of blood pressure using a sphygmomanometer are known as?
  1. Aminoff’s sounds
  2. Korotoff’s sounds
  3. Riva-Rocci sounds
  4. Hales’ sounds
3. During estimation of blood pressure, the sounds heard are produced due to?
  1. Increased diameter of vessel
  2. Increased viscosity of blood
  3. Increased velocity of blood flow
  4. None of the above
4. If the pressure in the arm cuff is more than systolic blood pressure, which of the following statements is true?
  1. A feeble pulse may be felt at the wrist
  2. A normal pulse may be felt at the wrist
  3. No pulse will be felt at the wrist
  4. None of the above
5. If the pressure in the arm cuff is less than diastolic blood pressure, which of the following statements is true?
  1. A feeble pulse may be felt at the wrist
  2. A normal pulse may be felt at the wrist
  3. No pulse will be felt at the wrist
  4. None of the above
6. Which of the following statements is true?
  1. When the pressure in the cuff is less than the diastolic blood pressure, turbulence will be produced
  2. Sounds may be heard when a stethoscope placed over any artery in the body
  3. Korotkoff’s sounds are heard as long as the cuff pressure stays between systolic and diastolic blood pressure
  4. None of the above
7. Which of the following methods gives the best estimate of blood pressure?
  1. Palpatory method
  2. Auscultatory method
  3. Direct method
  4. All the three methods give an equally reliable estimate of blood pressure
8. For correct estimation of blood pressure, the cuff should cover ___ of the arm circumference?
  1. One third
  2. Two third
  3. Half
  4. Any of the above
9. Which of the following statements is true?
  1. The cuff used for BP estimation should be highly compliant
  2. Auscultatory method is a more precise method of BP estimation than palpatory method
  3. Direct method for estimation of blood pressure is recommended for use in day to day practice
  4. All of the above
10. What is the formula used for calculating Mean blood pressure?
  1. DBP + 1/3 (PP)
  2. SBP + 1/3 (PP)
  3. SBP + 1/3 (DBP)
  4. DBP + 1/3 (SBP)
11. Diameter of inflatable rubber cuff for adults is
  1. 23 x 12.5 cm
  2. 32 x 12.5 cm
  3. 21 x 10.5 cm
  4. 25 x 13.5 cm
12. The following statement is true about *white coat hypertension*?
  1. Patient faints on seeing a doctor
  2. Patient’s blood pressure increases on seeing a doctor
  3. Patient’s blood pressure increases on wearing white clothes
  4. Patient’s heart rate decreases on seeing a doctor
13. Define auscultatory gap
  1. Absence of Phase I Korotkoff’s sounds
  2. Absence of Phase II Korotkoff’s sounds
  3. Disappearance of Korotkoff’s sounds between Phase I and II
  4. Complete absence of Korotkoff’s sounds
14. Name the labile component of blood pressure.
  1. Systolic
  2. Diastolic
  3. Mean
  4. Pulse
15. How will blood pressure recording be affected by an undersized cuff?
  1. Higher than normal
  2. Lower than normal
  3. Equal to normal
  4. Cannot be recorded using an undersized cuff

Appendix II: Qualitative Feedback

Q1. Please describe your experience of attending the demonstration of blood pressure measurement on a digital system.

Q2. How was the demonstration using the digital system different than that done using aneroid sphygmomanometer?

Q3. How can we improve the demonstration to make it more useful for students?

## References

[1] Podoll A, Grenier M, Croix B, Feig DI (2007). Inaccuracy in pediatric outpatient blood pressure measurement. Pediatrics.

[2] Pickering TG, Hall JE, Appel LJ, Falkner BE, Graves J, Hill MN, Jones DW, Kurtz T, Sheps SG, Roccella EJ (2005). Recommendations for blood pressure measurement in humans and experimental animals: part 1: blood pressure measurement in humans: a statement for professionals from the Subcommittee of Professional and Public Education of the American Heart Association Council on High Blood Pressure Research. Circulation.

[3] Gazibara T, Rancic B, Maric G, Radovanovic S, Kisic-Tepavcevic D, Pekmezovic T (2015). Medical students, do you know how to measure blood pressure correctly?. Blood Press Monit.

[4] Eghbalibabadi M, Ashouri E (2014). Comparison of the effects of two teaching methods on the nursing students’ performance in measurement of blood pressure. Iran J Nurs Midwifery Res.

[5] Brokalaki H, Matziou V, Gymnopoulou E, Galanis P, Brokalaki E, Theodossiades G (2008). Modification of nursing students' performance in blood pressure measurement: an educational retraining programme. Int Nurs Rev.

[6] Komine H, Sugawara J, Hayashi K, Yoshizawa M, Yokoi T (2009). Regular endurance exercise in young men increases arterial baroreflex sensitivity through neural alteration of baroreflex arc. J Appl Physiol (1985).

[7] Michael JA, Wenderoth MP, Modell HI, Cliff W, Horwitz B, McHale P, Richardson D, Silverthorn D, Williams S, Whitescarver S (2002). Undergraduates' understanding of cardiovascular phenomena. Adv Physiol Educ.

[8] Hodgson Y, Choate J (2012). Continuous and noninvasive recording of cardiovascular parameters with the Finapres finger cuff enhances undergraduate student understanding of physiology. Adv Physiol Educ.

[9] Bell FE 3rd, Wilson LB, Hoppmann RA (2015). Using ultrasound to teach medical students cardiac physiology. Adv Physiol Educ.

[10] Hammoudi N, Arangalage D, Boubrit L, Renaud MC, Isnard R, Collet JP, Cohen A, Duguet A (2013). Ultrasound-based teaching of cardiac anatomy and physiology to undergraduate medical students. Arch Cardiovasc Dis.

[11] Ribaric S, Kordas M (2011). Teaching cardiovascular physiology with equivalent electronic circuits in a practically oriented teaching module. Adv Physiol Educ.

[12] Paganini M, Rubini A (2016). Ultrasound-based lectures on cardiovascular physiology and reflexes for medical students. Adv Physiol Educ.

[13] Saleh AM, Shabila NP, Dabbagh AA, Al-Tawil NG, Al-Hadithi T (2015). A qualitative assessment of faculty perspectives of small group teaching experience in Iraq. BMC Med Educ.

[14] Biswas SS, Jain V, Agrawal V, Bindra M (2015). Small group learning: effect on item analysis and accuracy of self-assessment of medical students. Educ Health (Abingdon).

[15] Edmunds S, Brown G (2010). Effective small group learning: AMEE Guide No. 48. Med Teach.

[16] Parmelee D (2011). Effective small group learning: Guide supplement 48.1--viewpoint. Med Teach.

[17] Michael J (2006). Where's the evidence that active learning works?. Adv Physiol Educ.

